# No influence of the P-glycoprotein polymorphisms MDR1 G2677T/A and C3435T on the virological and immunological response in treatment naïve HIV-positive patients

**DOI:** 10.1186/1476-0711-4-3

**Published:** 2005-01-20

**Authors:** Ralf Winzer, Peter Langmann, Michael Zilly, Franz Tollmann, Jörg Schubert, Hartwig Klinker, Benedikt Weissbrich

**Affiliations:** 1 Medical Policlinic, Division of Infectious Diseases, University of Würzburg, Josef-Schneider-Str. 2, 97080 Würzburg, Germany; 2 Institute of Virology and Immunobiology, University of Würzburg, Versbacher-Str. 7, 97078 Würzburg, Germany

## Abstract

**Background:**

In a retrospective study of HIV-infected patients, we investigated the influence of the MDR1 genotype (G2677T/A and C3435T) on the virological and immunological response of treatment naïve patients.

**Methods:**

The MDR1 genotype was analysed from 72 patients in whom antiretroviral therapy was initiated between 1998 and 2004. Data were obtained at week 4, 12, 24 and 48 and were analysed by the Kruskal-Wallis test.

**Results:**

During the first 48 weeks of antiretroviral therapy, there were no significant differences in the virological and immunological response with respect to the MDR1 2677 and 3435 genotypes and the 2677/3435 haplotype.

**Conclusions:**

In view of different results from several studies concerning the influence of MDR1 polymorphisms on the immunological and virological response to antiretroviral therapy, further studies with larger patient groups and longer follow-up are necessary in order to resolve conflicting issues.

## Background

The P-glycoprotein (P-gp) is an ATP-dependent efflux transporter (ABCB1) encoded by the multidrug resistance gene (MDR1) which extrudes large lipophilic, positvely charged molecules from cells, among them HIV-1 protease inhibitors [1,2]. P-gp is expressed on a variety of cells including human lymphocytes, the target cells of HIV and of antiretroviral substances [3]. 48 single nucleotide polymorphisms (SNP) have been described so far for the MDR1 gene [4]. Though there is still controversy about the biological relevance of these SNPs, there appears to be an association between specific genotypes and mRNA expression, P-gp expression and/or P-gp function (reviewed in [5,6]). Recently, Fellay *et al*. found lower nelfinavir and efavirenz plasma levels associated with the TT genotype of the SNP C3435T in exon 26 and a greater rise of the CD4 cell count 6 months after initiation of antiretroviral therapy in patients with this genotype [7]. However, the mechanisms underlying this observation remain unclear. In contrast to protease inhibitors (PI), non-nucleoside reverse transcriptase inhibitors (NNRTI) such as efavirenz are not substrates of the P-gp. Therefore, it has been speculated that the P-gp may modulate the clinical course of HIV infection independent from its role in drug transport. Indeed, there have been reports showing inhibition of apoptosis and decreased HIV production in cells overexpressing P-gp [8-12]. However, these observations from in vitro studies have not been confirmed in vivo when the disease progression before treatment was assessed in HIV infected individuals with different MDR1 genotypes [13].

The genetic variant C3435T in exon 26 is a synonymous polymorphism that does not alter the amino acid sequence. How this variant could affect P-gp expression is still unknown [6]. According to one hypothesis, functional effects of the C3435T SNP may not be genotype- but haplotype-dependent. The exon 26 C3435T polymorphism is in linkage disequlibrium with the polymorphism G2677T in exon 21, which results in the amino acid change Ala893Ser [6]. 2677A leading to Ala893Thr is an infrequent third allel of this SNP. The results of several studies on the functional effects of mutations at position 2677 in exon 21 have shown conflicting results [6].

Because of the unresolved issues surrounding the potential effects of MDR1 polymorphisms and P-gp function in HIV infection, we investigated whether there was an association between the MDR1 polymorphisms 3435 and 2677 and the immunological response in HIV infected individuals after initiation of antiretroviral therapy.

## Methods

### Patients

Of the HIV 1 infected patients seen at the Department of Infectious Diseases of the University of Würzburg, Germany, 72 patients (18 women, 54 men; mean age 39.5 years, range: 26 – 59 years; 64 Caucasians, 5 African, 3 Asian), started antiretroviral therapy between 1998 and 2004. The therapy consisted of three nucleoside reverse transcriptase inhibitors (NRTI) (n = 12), two NRTIs plus at least one PI (n = 40; including RTV n = 26) or two NRTIs plus one NNRTI (n = 20). HIV load and CD4 cell count were determined four weeks after initiation of therapy and approximately every three months thereafter. In patients who received NNRTI or PI drugs as part of their therapy, plasma levels were monitored and adjusted to therapeutic drug levels when necessary as previously described [14,15]. The treatment was based on current international treatment guidelines [16], taking into account individual circumstances of each patient (e.g. known intolerabilities, side-effects of previous therapies, concomitant medication).

The study was in accordance with the Helsinki Declaration and was approved by the local ethics committee. Patients gave informed consent for the study.

### Genotype analysis

DNA was extracted from 200 μl blood and the MDR1 3435 genotype was determined with genotype specific hybridisation probes and melting curve analysis on the LightCycler (Roche, Mannheim, Germany) as previously described [17]. The 2677 genotype was determined in a similar fashion [18]. Briefly, DNA was amplified on the LightCycler with the Quantitect Probe PCR Kit (Qiagen, Hilden, Germany) by using the primers 5'-gcaggagttgttgaaatgaaaatg-3' (forward) and 5'-cgcctgctttagtttgactca-3' (reverse). Hybridization probes were added to the master mix to a final concentration of 0.05 μM (sensor probe: 5'-ttcccagTaccttct-fluorescein; locked nucleic acid base in upper case letter) and 0.15 μM (anchor probe: 5'-LC Red640-ctttcttatctttcagtgcttgtcc-p). Primers and probes were obtained from TIB Molbiol (Berlin, Germany). The melting points were 41°C, 47°C, and 52°C for the T-, G-, and A-alleles, respectively. The results of 30 samples were confirmed by sequencing.

### Statistical analysis

Data were analysed by the Kruskal-Wallis test, which as a nonparametric test compairs three or more unpaired groups. Because of the small number of samples a nonparametric test was needed. SPSS version 12.0 (SPSS GmbH, Munich, Germany) was used for statistical analysis. A *P*-value of 0.05 was considered to be significant. Analogous to [13], the SNPs 3435 and 2677 were analyzed both separately and in combination as composite genotypes: H1: 2677GG and 3435CC (wild type); H2: 2677GT or TT and 3435CT or TT (2677T/3435T haplotype carrier); H3: 2677GG and 3435CT or TT; H4: 2677GT or TT and 3435CC; and H5: 2677AG or AT and any 3435 genotype (2677A carrier).

## Results

At initiation of antiretroviral therapy 8 patients had viral loads of <10.000 copies/ml, 24 patients between >10.000 and <100.000 copies/ml, and 40 patients >100.000 copies/ml. 39 patients had a CD4 cell count of <200 cells/μl, 16 patients between >200 and <350 cells/μl, and 17 patients >350 cells/μl. Determination of NNRTI- and PI drug levels indicated compliance of the patients with a NNRTI or PI containing regimen, because the measured drug levels were in the therapeutic range. Genotype analysis of the MDR1 gene at position 3435 in exon 26 revealed 20 patients with the CC genotype, 33 with the CT genotype and 19 with the TT genotype (5 patients of African origin: 4 with CC and 1 with CT genotype; 3 patients o f Asisan origen: 2 with CT and 1 with TT genotye). Analysis of the 2677 polymorphism in exon 21 demonstrated that 24 patients had the GG-, 23 the GT-, 18 the TT-, 4 the AG-, and 3 the AT genotype; the AA genotype was not found in this group (5 patients of African origin: 4 with GG and 1 with GT genotype; 3 patients of Asian origin: 3 with TT genotype). Detailed genotype and haplotype results with respect to the initial viral load and CD4 cell count are presented in table 1.

**Table 1:** Genotype analysis of 72 treatment naive HIV-positive patients with respect to baseline CD4 cell count and viral load.

CD4 [cells/μl]	VL [copies/μl]	n =	2677						3435			2677/3435^$^				
			
			GG	GT	TT	AG	AT	AA	CC	CT	TT	H1	H2	H3	H4	H5
<200	>10^5^	27	7	7	8	3	2	-	7	10	10	5	15	2	-	5
	10^4 ^– 10^5^	8	4	3	1	-	-	-	2	6	-	2	4	2	-	-
	<10^4^	4	2	1	1	-	-	-	1	2	1	1	2	1	-	-

200	>10^5^	6	2	3	1	-	-	-	1	4	1	1	4	1	-	-
-350	10^4 ^– 10^5^	9	3	3	3	-	-	-	3	3	3	3	6	-	-	-
	<10^4^	1	1	-	-	-	-	-	1	-	-	1	-	-	-	-

>350	>10^5^	7	1	4	2	-	-	-	1	3	3	1	6	-	-	-
	10^4 ^– 10^5^	7	1	2	2	1	1	-	2	4	1	1	4	-	-	2
	<10^4^	3	3	-	-	-	-	-	2	1	-	2	-	1	-	-

*total*		*72*	*24*	*23*	*18*	*4*	*3*	*0*	*20*	*33*	*19*	*17*	*41*	*7*	-	*7*

^$^composite genotypes: H1: 2677GG and 3435CC (wild type); H2: 2677GT or TT and 3435CT or TT (2677T/3435T haplotype carrier); H3: 2677GG and 3435CT or TT; H4: 2677GT or TT and 3435CC; and H5: 2677AG or AT and any 3435 genotype (2677A carrier)

**Figure 1:**
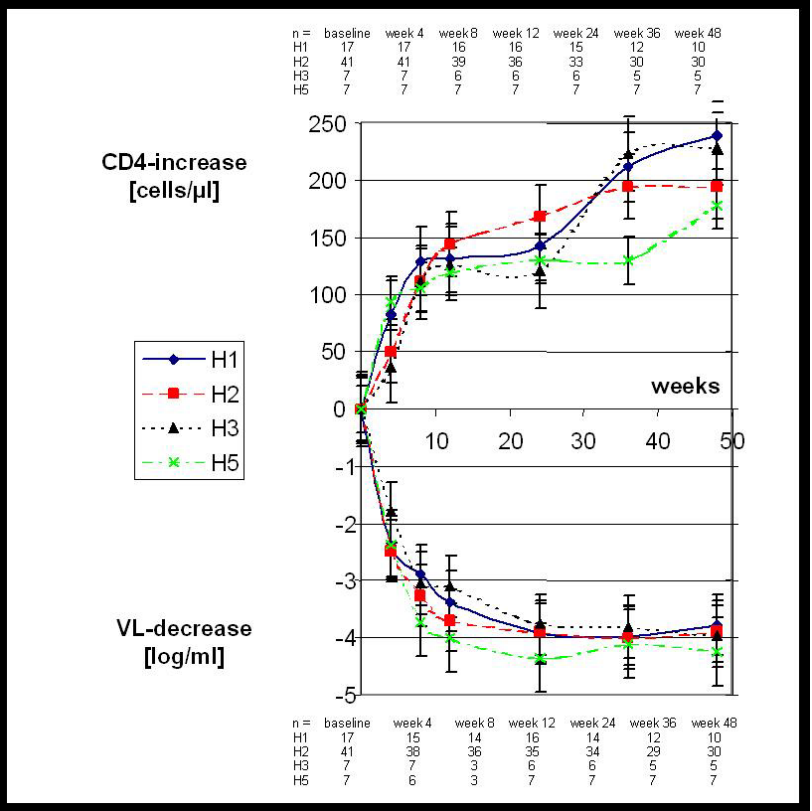
Composite genotypes (see methods) and response to antiretroviral treatment. Suppression of viraemia (lower panel) and CD4-cell count increase (upper panel) are shown. Data are mean values ± standard error.

Table 2 and 3 show the median and mean values of the viral load decline and the CD4-cell increase, respectively, determined at week 4, 12, 24 and 48 after initiation of therapy. There were no significant differences of the viral load decline neither between patient groups with different genotypes at positions 2677 or 3435 nor between patient groups with different 2677/3435 haplotypes. As to the CD4-cell response, there were no significant differences between the different genotypes and haplotypes, either. There was a trend of a more pronounced mean CD4-cell increase at week 12 and 24 in patients with the 3435TT genotype. However, this trend did not persist at week 48 and was not confirmed by the corresponding median values. A graphical presentation of the mean viral load decrease and CD4-cell increase with respect to the 2677/3435 haplotypes is given in fig. 1. Analysis of patients with different therapy-regimens (only NRTI, NRTI + NNRTI, or NRTI + PI) revealed no differences in the virological and immunological response between the different genotypes either, but was limited by small patient numbers in the subgroups (data not shown).

**Table 2:** VL-decrease [log copies/ml] at week 4, 12, 24 and 48 after initiation of antiretroviral therapy with respect to the MDR1 2677 and 3435 genotypes. Statistical analysis was done with the Kruskall-Wallis test.

		week 4					week 12					week 24					week 48				
		
MDR1		n =	median [log/ml]	mean [log/ml]	SA	*p *=	n =	median [log/ml]	mean [log/ml]	SA	*p *=	n =	median [log/ml]	mean [log/ml]	SA	*p *=	n =	median [log/ml]	mean [log/ml]	SA	*p *=
2677	GG	22	-2,301	-2,210	0,590	*>0.3*	22	-3,048	-3,287	0,682	*>0.05*	20	-3,943	-3,876	0,748	*>0.9*	15	-3,716	-3,835	0,777	*>0.5*
	GT	21	-2,602	-2,537	0,566		19	-3,786	-3,794	0,635		17	-3,929	-3,933	0,499		16	-3,942	-3,946	0,488	
	TT	17	-2,495	-2,372	0,651		16	-3,739	-3,602	0,595		17	-4,000	-3,850	0,708		14	-3,977	-3,795	0,615	
	AG	3	-2,301	-2,689	0,606	§	4	-3,952	-4,167	0,884	§	4	-4,500	-4,537	0,702	§	4	-4,151	-4,362	0,726	§
	AT	3	-1,875	-2,032	0,493	§	3	-4,176	-3,752	0,690	§	3	-4,176	-4,085	0,223	§	3	-4,176	-4,085	0,223	§

3435	CC	17	-2,301	-2,382	0,501	*>0.6*	19	-3,301	-3,416	0,699	*>0.4*	17	-4,000	-3,974	0,685	*>0.7*	13	-3,716	-3,818	0,709	*>0.6*
	CT	32	-2,247	-2,363	0,640		29	-3,778	-3,742	0,737		28	-3,812	-3,830	0,704		25	-3,929	-3,984	0,614	
	TT	17	-2,398	-2,348	0,668		17	-3,602	-3,530	0,595		17	-4,176	-4,054	0,571		15	-4,114	-3,849	0,609	

2677/3435^†^	H1	15	-2,398	-2,398	0,531	*>0.8*	16	-3,199	-3,361	0,724	*>0.08*	14	-3,943	-3,926	0,715	*>0.8*	10	-3,659	-3,774	0,787	*>0.4*
	H2	38	-2,548	-2,463	0,611		35	-3,778	-3,706	0,624		34	-3,954	-3,891	0,614		30	-3,954	-3,875	0,556	
	H3	7	-1,778	-1,806	0,502	§	6	-2,827	-3,089	0,506	§	6	-3,827	-3,761	0,807	§	5	-4,176	-3,958	0,741	§
	H5	6	-2,261	-2,361	0,642	§	7	-4,176	-3,989	0,833	§	7	-4,301	-4,343	0,594	§	7	-4,176	-4,243	0,584	§

total		66					64					61					52				

^†^composite genotypes: see methods; ^§^excluded from statistical analysis

**Table 3:** CD4-cell increase [cells/μl] at week 4, 12, 24 and 48 after initiation of antiretroviral therapy with respect to the MDR1 2677 and 3435 genotypes. Statistical analysis was done with the Kruskall-Wallis test.

		week 4					week 12					week 24					week 48				
		
MDR1		n =	median [cells/μl]	mean [cells/μl]	SA	*p *=	n =	median [cells/μl]	mean [cells/μl]	SA	*p *=	n =	median [cells/μl]	mean [cells/μl]	SA	*p *=	n =	median [cells/μl]	mean [cells/μl]	SA	*p *=
2677	GG	24	56,5	69,8	81,5	*>0.3*	22	97,0	130,6	132,4	*>0.5*	21	107,0	136,1	120,5	*>0.8*	15	244,0	235,7	132,1	*>0.1*
	GT	23	24,0	37,8	82,5		20	84,5	144,3	157,2		17	132,0	180,8	178,0		16	161,5	238,3	218,6	
	TT	18	9,5	67,4	117,7		16	70,0	143,9	222,3		16	95,0	154,5	162,0		14	107,5	143,9	159,1	
	AG	4	70,5	85,8	68,5	§	4	82,5	74,8	34,4	§	4	54,5	80,3	81,3	§	4	131,5	126,5	71,0	§
	AT	3	112,0	105,7	13,4	§	3	176,0	180,7	63,8	§	2	154,5	154,5	54,5	§	3	172,0	248,0	118,2	§

3435	CC	20	76,0	84,8	78,6	>0.1	19	91,0	123,0	113,0	>0.9	18	106,5	132,4	112,4	>0.5	13	191,0	220,1	121,5	>0.6
	CT	34	25,5	47,2	76,1		30	85,0	123,7	135,3		28	131,0	134,2	106,8		25	159,0	187,7	118,4	
	TT	18	12,0	61,6	119,1		17	74,0	175,9	228,1		16	134,0	212,2	214,7		15	122,0	223,9	259,3	

2677/3435^†^	H1	17	72,0	83,1	78,7	*>0.1*	16	115,0	132,1	119,8	*>0.8*	15	107,0	142,2	113,3	*>0.9*	10	194,0	239,6	128,4	*>0.5*
	H2	41	20,0	50,8	100,6		36	74,5	144,1	188,9		33	130,0	168,1	170,9		30	134,5	194,2	198,8	
	H3	7	19,0	37,3	79,1	§	6	92,0	126,7	161,2	§	6	122,0	120,8	135,7	§	5	302,0	227,8	138,9	§
	H5	7	87,0	94,3	53,5	§	7	105,0	120,1	71,9	§	7	100,0	131,0	98,7	§	7	172,0	178,6	111,7	§

*total*		72					65					61					52				

^†^composite genotypes: see methods; ^§^excluded from statistical analysis

## Discussion

In an analysis of the virological and immunological response of treatment naïve patients with respect to the MDR1 G2677T/A and C3435T polymorphisms, virus load and CD4 cell count were assessed longitunally after initiation of antiretroviral therapy. We did not find an association between the CD4-cell increase or the HIV load decline and the MDR1 2677 or 3435 genotype during the first year of therapy.

Our data are in contrast to a report of Fellay *et al*. that showed a significantly greater mean CD4-cell rise in patients with the MDR1 3435TT genotype during an observation period of 24 weeks [7]. The number of patients in the previous report (n = 96) was slightly larger than our population (n = 72), but the number of homozygotes of the 3435 polymorphism was very similar (22CC/20TT in [7], 20CC/19TT this study). Though we saw a trend towards a greater mean CD4-cell rise in patients with the 3435TT genotype as well, we doubt that this is indication of a real difference, because this trend was not seen when median values were considered. In our study, neither median values nor mean values were significantly different by Kruskal-Wallis test and ANOVA, respectively. For genotype subgroup sizes as in this study, analysis of the mean values is probably less robust. If there was a real difference between the immunological response of patients with the 3435CC and TT genotype, it did not persist at week 48. At this time point, mean and median values in the two groups were almost identical. A possible explanation for the diverse results is the selection of the study patients. While we included all treatment naïve patients in whom therapy was started between 1998 and 2004, Fellay *et al*. selected part of the patients on the basis of long-term viral suppression. In both studies, a variety of antiretroviral regimens was used. While all of the patients in the study of Fellay *et al*. received either nelfinavir or efavirenz, the treatment regimens in our study were more heterogenous and did not allow a meaningful separate analysis.

The results of our study are supported by Nasi *et al*. [19] who analysed data of 149 treatment naïve patients who were treated with a PI-containing regimen (n = 106) or a NNRTI-containing regimen (n = 46) and found no association between the MDR1 genotype at position 3435 and the CD4 cell increases or plasma viral load decreases during the first six months of treatment among individuals with different genotypes. Likewise, in 142 patients enrolled in an open-label, randomized phase IIIb study comparing nelfinavir and efavirenz for treatment of HAART-naïve individuals the CD4 cell counts did not increase to a higher level in individuals with the homozygous variant genotype (TT) at the MDR1 C3435T locus in either the nelfinavir or the efavirenz treatment groups [20].

The MDR1 3435 polymorphism is a synonymous polymorphism. The TT genotype may have a reduced translation efficiency or lead to post-translational differences [21-23]. It has been shown, that a linkage disequilibirum exists between the exon 26 C3435T and the exon 21 G2677T/A polymorphism [6]. Therefore, we investigated both SNPs in our study. Neither the 2677 polymorphism alone nor the 2677/3435 haplotype was associated with differences in the virological or immunological response of treatment naïve patients. These data are in agreement with those of Haas *et al*. [24], who showed that the phase 1 and phase 2 viral decay as well as changes in CD4- and CD8-T-cells during triple therapy with ritonavir, zidovudine, and lamivudine in 31 treatment naïve patients were not associated with the MDR1 2677 and 3435 genotypes.

In a retrospective study of 455 treatment naïve patients initiating antiretroviral therapy with 40 months of follow-up [25], there was a trend to earlier virological failure in the 3435CC group (*p *= 0.07) with no effect of the C3435T polymorphism in the MDR-1 gene on immunological failure. However, the difference in the virological response was not observed during the first 10 months. Further follow-up of our patient group is ongoing in order to detect long-term effects that may not have been apparent during the observation period analysed in this report.

## Conclusions

During a follow-up of 48 weeks, we found no evidence for an association between the MDR1 G2677T/A and C3435T polymorphisms and the virological and immunological response to therapy in HIV-positive drug-naïve patients. The individual response to antiretroviral therapy is a complex phenomenon, which is influenced by a large number of biological variables. Further studies on the role of polymorphisms of the MDR1 and other transporters and enzymes involved in drug metabolism are necessary in order to elucidate the role of pharmacogenetic effects in HIV therapy.

## Competing interests

None declared.

## Authors' contributions

RW participated in the design of the study and the molecular genetic studies, performed the statistical analysis and drafted the manuscript. PL participated in the design of the study, obtained and reviewed clinical data and helped in the data analysis. MZ participated in the clinical management of the study patients. FT and JS participated in the molecular genetic studies and the virological and immunological analysis. HK participated in the study design and coordinated clinical management of the study patients. BW participated in the study design and coordination and made contributions to the manuscript. All authors read and approved the final manuscript.
